# Alpha‐Lipoic Acid Reduces NLRP3/ASC Expression and IL‐1β Release in Kupffer Cells and Improves Insulin Signaling in FL83B Hepatocytes Exposed to a Conditioned Medium

**DOI:** 10.1002/fsn3.71517

**Published:** 2026-02-03

**Authors:** Chih‐Yuan Ko, Yangming Martin Lo, Thi Kim Ngan Nguyen, Shao‐Ting Kao, Chung‐Hsin Wu, Wen‐Chung Huang, Szu‐Chuan Shen

**Affiliations:** ^1^ Department of Clinical Nutrition The Second Affiliated Hospital of Fujian Medical University Quanzhou China; ^2^ School of Public Health Fujian Medical University Fuzhou China; ^3^ College of Sustainability National Tsing Hua University Hsinchu Taiwan; ^4^ Graduate Program of Nutrition Science National Taiwan Normal University Taipei Taiwan; ^5^ School of Life Science National Taiwan Normal University Taipei Taiwan; ^6^ Graduate Institute of Health Industry Technology Chang Gung University of Science and Technology Taoyuan Taiwan

**Keywords:** alpha‐lipoic acid, diabetes mellitus, FL83B hepatocytes, Kupffer cells, NLRP3 inflammasome

## Abstract

Type 2 diabetes mellitus (T2DM) is characterized by insulin resistance and chronic inflammation. This study investigated whether alpha‐lipoic acid (ALA), a redox‐active compound with established anti‐inflammatory properties, can inhibit the activation of the nucleotide‐binding oligomerization domain‐like receptor family pyrin domain‐containing 3 (NLRP3) inflammasome in lipopolysaccharide (LPS)‐stimulated Kupffer cells and mitigate inflammation‐induced insulin resistance in FL83B hepatocytes. Kupffer cells were pretreated with ALA prior to exposure to LPS and either adenosine triphosphate or nigericin to activate NLRP3 inflammasome. The resulting conditioned medium was collected for cytokine analysis and subsequently used to treat FL83B hepatocytes. ALA reduced LPS‐induced interleukin‐1β (IL‐1β) secretion in a concentration‐dependent manner, whereas a modest but significant decrease in tumor necrosis factor‐alpha (TNF‐α) was observed only at the highest dose (2000 μM; *p* < 0.05). Western blot analysis demonstrated that ALA suppressed the expression of NLRP3 and nuclear factor‐kappa B (NF‐κB) (*p* < 0.05) and inhibited the phosphorylation of extracellular signal‐regulated kinase (ERK). Additionally, ALA preserved mitochondrial membrane potential in Kupffer cells. Kupffer cells treated with ALA (100 μM) prior to LPS stimulation significantly enhanced glucose uptake and upregulated the expression of insulin signaling related proteins, including phosphorylated phosphoinositide 3‐kinase (p‐PI3K), phosphorylated protein kinase B (p‐Akt) and glucose transporter type 2 (GLUT2) expression, in FL83B hepatocytes cultured with a conditioned medium from LPS‐primed and ATP/nigericin‐stimulated Kupffer cells (*p* < 0.05). These findings highlight the potential of ALA as a modulator of hepatic immune‐metabolic interactions and support its therapeutic relevance for managing insulin resistance in T2DM.

## Graphical abstract

Alpha‐lipoic acid (ALA) reduced NLRP3/ASC expression, attenuated upstream NF‐κB/ERK signaling, and decreased IL‐1β release while preserving mitochondrial membrane potential in murine Kupffer cells subjected to LPS priming and a second signal. Conditioned medium from ALA‐pretreated Kupffer cells (particularly 100 μM) enhanced glucose uptake and improved insulin signaling (p‐PI3K, p‐Akt, and GLUT2) in FL83B hepatocytes. Together, these findings indicate that ALA modulates hepatic immune‐metabolic crosstalk and may help counteract inflammation‐associated insulin resistance in type 2 diabetes.

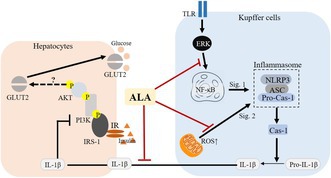

## 1 Introduction

Type 2 diabetes mellitus (T2DM) has become an increasingly important global health issue. In 2021, approximately 537 million individuals were living with T2DM, and this number is projected to increase to 783 million by the year 2045 (International Diabetes Federation 2021). T2DM is primarily characterized by insulin resistance, a pathological condition in which target tissues exhibit a reduced response to insulin, a hormone essential for glucose regulation and uptake (Ahmad et al. 2022). Persistent hyperglycemia resulting from insulin resistance imposes metabolic stress on pancreatic beta cells, often culminating in their dysfunction and the onset of multiple metabolic complications (Ahlqvist et al. 2021).

A growing body of evidence supports the notion that oxidative stress and chronic low‐grade inflammation are fundamental contributors to the pathogenesis of insulin resistance (Juzbašić et al. 2025; Rains and Jain 2011). Mitochondrial dysfunction, frequently resulting from metabolic overload, leads to excessive production of reactive oxygen species (ROS), which can impair insulin signaling through oxidative modifications of proteins and lipids (Rains and Jain 2011). In the liver, Kupffer cells, which are resident macrophages, play an essential role in the modulation of innate immune responses (Xu et al. 2023). Upon stimulation with lipopolysaccharide (LPS), Kupffer cells initiate intracellular signaling cascades that promote the formation of inflammasomes, which are cytosolic multiprotein complexes involved in inflammatory regulation (Jo et al. 2016). Among them, the nucleotide‐binding oligomerization domain‐like receptor family pyrin domain‐containing 3 (NLRP3) inflammasome is particularly important, as it regulates the maturation of interleukin‐1 beta (IL‐1β), a cytokine that has been demonstrated to disrupt hepatic insulin signaling and aggravate insulin resistance (Kim et al. 2016; Meier et al. 2025).

Alpha‐lipoic acid (ALA) is a naturally occurring disulfide compound found in various animal‐ and plant‐based foods. It is mainly present as protein‐bound lipoyllysine in metabolically active animal tissues such as kidney (2.64 μg/g), heart (1.51 μg/g), liver (0.86 μg/g), skeletal muscle (0.97 μg/g), and in vegetables such as spinach (3.15 μg/g), potato (3.09 μg/g), broccoli (0.94 μg/g), and tomato (0.56 μg/g) on a dry‐weight basis (Choudhary et al. 2023). ALA serves as a cofactor for mitochondrial oxidative decarboxylation and exhibits a range of pharmacological properties, including antioxidant, anti‐inflammatory, and insulin‐sensitizing effects (Wang et al. 2025). ALA promotes cellular redox balance by regenerating endogenous antioxidants such as glutathione, vitamin C, and vitamin E (Rochette et al. 2013). Due to its amphipathic nature, ALA is able to interact with both aqueous and lipid environments, which contributes to its widespread tissue bioavailability (Salehi et al. 2019). Research has indicated that ALA enhances glucose metabolism in insulin‐resistant cells by activating the phosphoinositide 3‐kinase (PI3K) and protein kinase B (AKT) signaling pathways (Ko, Lo, et al. 2021; Ko et al. 2023; Awoleye and Adedeji 2024). Furthermore, ALA has been shown to increase the expression and membrane translocation of glucose transporters such as GLUT2 and GLUT4, both of which are necessary for effective glucose uptake (Konrad et al. 2001; Salehi et al. 2019; Sztolsztener and Chabowski 2024).

Our previous investigations have highlighted the therapeutic effects of ALA in animal models of T2DM. In rats with diet‐induced and streptozotocin‐induced diabetes, treatment with ALA significantly reduced hepatic triglyceride levels and suppressed the expression of proteins associated with inflammasome activity, including NLRP3, apoptosis‐associated speck‐like protein containing a caspase recruitment domain (ASC), caspase‐1, and IL‐1β (Ko, Lo, et al. 2021). ALA also alleviated skeletal muscle atrophy by modulating the activity of tumor necrosis factor alpha (TNF‐α), c‐Jun N‐terminal kinase (JNK), and the PI3K signaling pathway (Ko et al. 2023). Additional studies demonstrated that ALA improved hippocampal insulin signaling and cognitive performance in diabetic rats (Ko, Xu, et al. 2021), and enhanced amyloid beta clearance in microglial cells, suggesting broader implications for neuroimmune regulation (Ko et al. 2022). Although these systemic benefits are well established, the specific effect of ALA on inflammasome activation in hepatic immune cells remains poorly understood. In particular, there is limited evidence regarding how Kupffer cell‐derived inflammatory factors influence insulin signaling in hepatocytes. Most studies have focused on systemic metabolic outcomes without specifically addressing intrahepatic immune‐metabolic interactions.

In order to investigate these mechanisms, we employed an in vitro dual‐cell culture system consisting of immortalized murine Kupffer cells and FL83B hepatocytes. The primary aim of this study was to assess whether ALA pretreatment could attenuate LPS‐induced inflammasome activation in Kupffer cells, and whether the conditioned medium from these cells could improve insulin signaling in hepatocytes. We hypothesized that ALA regulates hepatic metabolic inflammation by suppressing NLRP3 inflammasome activity in Kupffer cells and enhancing hepatocyte insulin sensitivity through modulation of paracrine inflammatory signaling.

## 2 Materials and Methods

### 2.1 Cell Lines and Culture Conditions

Immortalized mouse Kupffer cells were obtained from Applied Biological Materials Inc. (Richmond, British Columbia, Canada). These cells were maintained in a culture medium composed of Prigrow II and Roswell Park Memorial Institute (RPMI) 1640 medium in a ratio of 4 to 1. The medium was supplemented with 10% fetal bovine serum (FBS), 1% L‐glutamine, and 1% penicillin–streptomycin. FL83B hepatocytes of murine origin were purchased from the American Type Culture Collection (ATCC, Manassas, Virginia, United States) and grown in F‐12 K nutrient mixture, a modified version of Ham's F‐12 medium, supplemented with 10% FBS. All cells were incubated at 37°C in a humidified atmosphere containing 5% carbon dioxide.

### 2.2 Reagents

ALA, LPS, nigericin, adenosine triphosphate (ATP), and additional chemical reagents were acquired from Sigma‐Aldrich (St. Louis, Missouri, United States). ALA was initially dissolved in dimethyl sulfoxide (DMSO) to create a concentrated stock solution and was subsequently diluted with cell culture medium to achieve working concentrations of 5, 100, 500, and 2000 μM immediately before use. The selective inhibitor of the NLRP3 inflammasome, MCC950, was reconstituted in sterile water to a stock concentration of 1172.5 μM. Additional reagents, including JC‐1 dye, 3‐(4,5‐dimethylthiazol‐2‐yl)‐2,5‐diphenyltetrazolium bromide (MTT), and insulin, were also purchased from Sigma‐Aldrich and used in accordance with the manufacturer's recommended procedures.

### 2.3 Cell Viability Assay

The MTT assay was employed to assess cell viability following ALA exposure. Kupffer cells were seeded in 96‐well culture plates at a density of 1 × 10^5^ cells per well and treated with various concentrations of ALA for 24 h. After treatment, 0.5 mg per milliliter of MTT reagent was added to each well and incubated for 4 h. Formazan crystals formed during the assay were dissolved in DMSO, and absorbance was measured at 570 nm using a microplate reader. The viability of cells was expressed as a percentage relative to untreated controls, following the method described by Xu et al. (2020).

### 2.4 NLRP3 Inflammasome Activation

To investigate activation of the NLRP3 inflammasome, Kupffer cells were preincubated with ALA (ranging from 5 to 2000 μM) or with MCC950 (20 μM) for 6 h, followed by stimulation with LPS (1 microgram per milliliter) for an additional 6 h. Subsequently, the cells were exposed to either nigericin (13.4 μM) or ATP (2 μM) for 1 h to induce inflammasome assembly. Cell culture supernatants were collected for determination of IL‐1β and tumor necrosis factor‐alpha (TNF‐α) concentrations using enzyme‐linked immunosorbent assay (ELISA) kits. Meanwhile, cell lysates were collected for immunoblot analysis to examine protein expression levels of NLRP3, ASC, and caspase‐1, according to previously published protocols (Ko et al. 2018; Ko, Lo, et al. 2021).

### 2.5 Insulin Resistance in FL83B Hepatocytes

To explore the influence of Kupffer cell‐derived inflammatory mediators on hepatocyte insulin sensitivity, FL83B cells were incubated with conditioned media collected from LPS‐primed/ATP‐stimulated Kupffer cells, with or without ALA pretreatment, for a duration of 24 h. After this incubation period, FL83B hepatocytes were stimulated with 1 μM insulin for 30 min. Protein expression levels of insulin signaling markers, including phosphorylated insulin receptor (p‐IR), phosphorylated phosphoinositide 3‐kinase (p‐PI3K), phosphorylated protein kinase B (p‐AKT), and glucose transporter 2 (GLUT2), were analyzed using Western blotting, following the procedures outlined in Xu et al. (2020).

### 2.6 Western Blot Analysis

The procedures for protein extraction and immunoblotting were performed in accordance with previously validated methods (Ko et al. 2022). Cells were lysed using an ice‐cold lysis buffer containing protease and phosphatase inhibitors. Protein concentrations were determined using the bicinchoninic acid (BCA) method. Equal amounts of protein (30 micrograms per lane) were separated by sodium dodecyl sulfate polyacrylamide gel electrophoresis and transferred onto polyvinylidene difluoride membranes. Membranes were then blocked with 5% non‐fat milk or bovine serum albumin and probed with specific primary antibodies, followed by incubation with horseradish peroxidase‐conjugated secondary antibodies. Protein bands were visualized using enhanced chemiluminescence and quantified using ImageJ software.

### 2.7 Mitochondrial Membrane Potential Assay

To assess mitochondrial membrane potential (MMP), JC‐1 staining was performed on Kupffer cells following treatment. Cells were incubated with JC‐1 dye at a concentration of 5 micrograms per milliliter at 37°C for 30 min. Fluorescent images were captured using a fluorescence microscope. A decline in the ratio of red to green fluorescence intensity was interpreted as indicative of mitochondrial depolarization, in accordance with the protocol described by Wang et al. (2019).

### 2.8 Glucose Uptake Assay

The capacity of FL83B hepatocytes to take up glucose was evaluated using the fluorescent glucose analog 2‐[N‐(7‐nitrobenz‐2‐oxa‐1,3‐diazol‐4‐yl)amino]‐2‐deoxy‐D‐glucose (2‐NBDG), as described in Xu et al. (2020). FL83B cells were seeded in 6‐well plates at a density of 3 × 10^5^ cells per well. After cell adhesion was established, cultures were treated for 24 h with conditioned medium diluted 9 to 1 with F‐12 K medium. Cells were subsequently washed with phosphate‐buffered saline, detached using trypsin, and resuspended in Krebs‐Ringer bicarbonate buffer supplemented with 1 μM insulin and 3 μM 2‐NBDG. Incubation was conducted in the dark at 37°C for 30 min. After centrifugation, the cells were resuspended in phosphate‐buffered saline and subjected to flow cytometric analysis using excitation at 488 nm and emission at 542 nm.

### 2.9 Statistical Analysis

All results were expressed as mean ± standard deviation. Statistical analyses were performed using SPSS software (version 23.0, IBM Corp., Armonk, NY, USA). One‐way analysis of variance (ANOVA) was used to compare group means, followed by Tukey's post hoc tests. A *p*‐value less than 0.05 was considered statistically significant.

## 3 Results

### 3.1 Effect of ALA on Viability of Kupffer Cells

**FIGURE 1:**
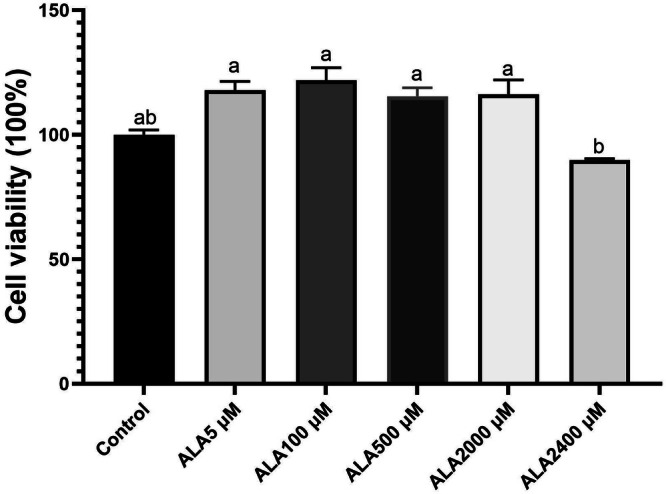
Effect of alpha‐lipoic acid (ALA) on Kupffer cells viability. Data are presented as mean ± SD, *n* = 4 for each group. a‐c letter is significantly different among all samples tested (*p* < 0.05).

The effect of ALA on Kupffer cell viability was assessed using the MTT assay. All tested concentrations of ALA (5–2400 μM), as referenced in Kiemer et al. (2002), maintained cell viability above 100%. No cytotoxic effects were observed at any concentration tested (Figure 1). However, due to solubility limitations at 2400 μM, the concentrations of 5, 100, 500, and 2000 μM were selected for subsequent experiments. To further ensure experimental validity, the cytotoxicity of MCC950 was also evaluated. All tested concentrations preserved cell viability above 90%, confirming that MCC950 did not compromise Kupffer cell viability under the experimental conditions (data not shown).

To evaluate the effects of ALA on NLRP3 inflammasome activation, Kupffer cells were pretreated with ALA at concentrations of 5, 100, 500, or 2000 μM prior to stimulation with lipopolysaccharide. Western blot analysis revealed that lipopolysaccharide significantly increased the expression levels of NLRP3, ASC, caspase‐1, and interleukin‐1 beta (IL‐1β) compared with untreated controls (*p* < 0.001). Pretreatment with ALA at 100, 500, and 2000 μM significantly suppressed lipopolysaccharide‐induced expression of these inflammasome‐related proteins in a concentration‐dependent manner. The 5 μM treatment exhibited a modest, non‐significant reduction.

### 3.2 ALA Suppresses LPS‐Induced IL‐1β Secretion in Kupffer Cells

**FIGURE 2:**
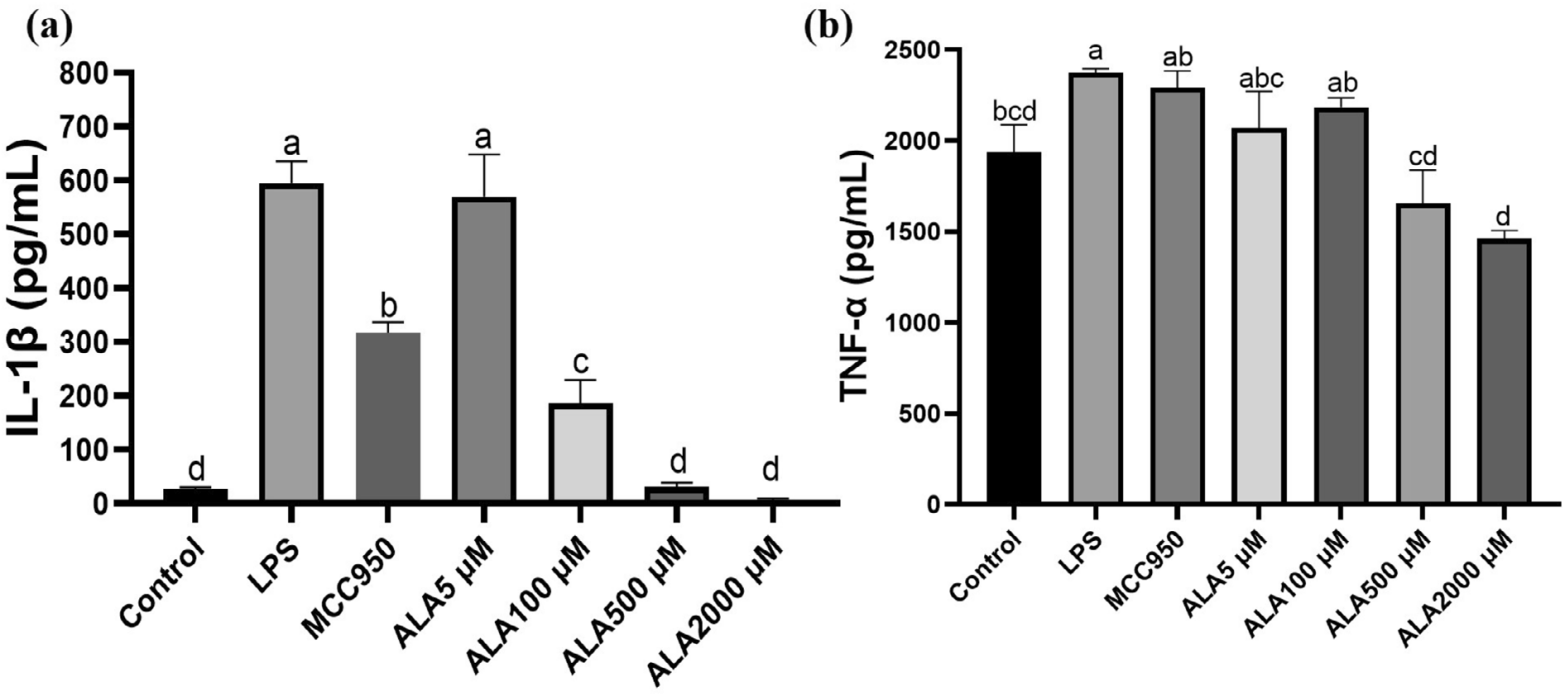
Effect of alpha‐lipoic acid (ALA) on IL‐1β (a) and TNF‐α (b) secretion in lipopolysaccharide (LPS)‐stimulated Kupffer cells. Data are presented as mean ± SD, *n* = 4 for each group. a‐d letter is significantly different among all samples tested (*p* < 0.05).

Stimulation of Kupffer cells with LPS resulted in a substantial increase in IL‐1β secretion, with a 21.9‐fold elevation compared to the untreated control group. Pretreatment with ALA at 2000 μM significantly suppressed IL‐1β secretion, resulting in an approximately 88% reduction (*p* < 0.05) (Figure 2a). A similar anti‐inflammatory effect was observed for TNF‐α, as ALA at the same concentration significantly reduced TNF‐α levels in LPS‐primed/ATP‐stimulated Kupffer cells (*p* < 0.05) (Figure 2b). These findings highlight the potent anti‐inflammatory properties of ALA in Kupffer cells.

### 3.3 ALA Maintaining Mitochondrial Health in Kupffer Cells

**FIGURE 3:**
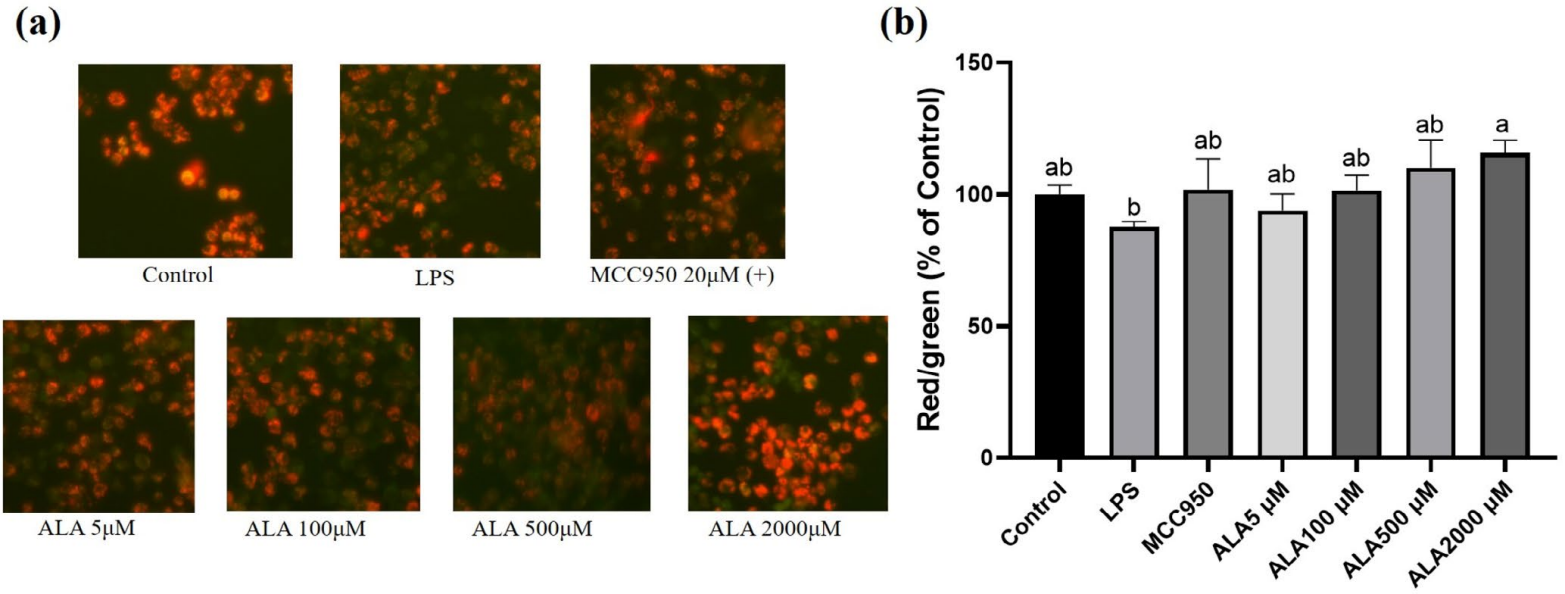
Effect of alpha‐lipoic acid (ALA) on mitochondrial membrane potential in lipopolysaccharide (LPS)‐stimulated Kupffer cells by JC‐1 staining (a) and quantification (b). Data are presented as mean ± SD, *n* = 3 for each group. a‐b letter is significantly different among all samples tested (*p* < 0.05).

To evaluate whether ALA preserves mitochondrial integrity, JC‐1 staining was used to assess MMP. LPS stimulation led to a significant decrease in the red‐to‐green fluorescence intensity ratio, indicative of mitochondrial depolarization. In contrast, pretreatment with ALA at 2000 μM effectively preserved MMP, as demonstrated by a higher red‐to‐green fluorescence ratio compared to the LPS‐treated group (Figure 3). These results suggest that ALA mitigates LPS‐induced mitochondrial dysfunction in Kupffer cells.

### 3.4 ALA Modulates Inflammasome‐Associated Protein Expression in Kupffer Cells

**FIGURE 4:**
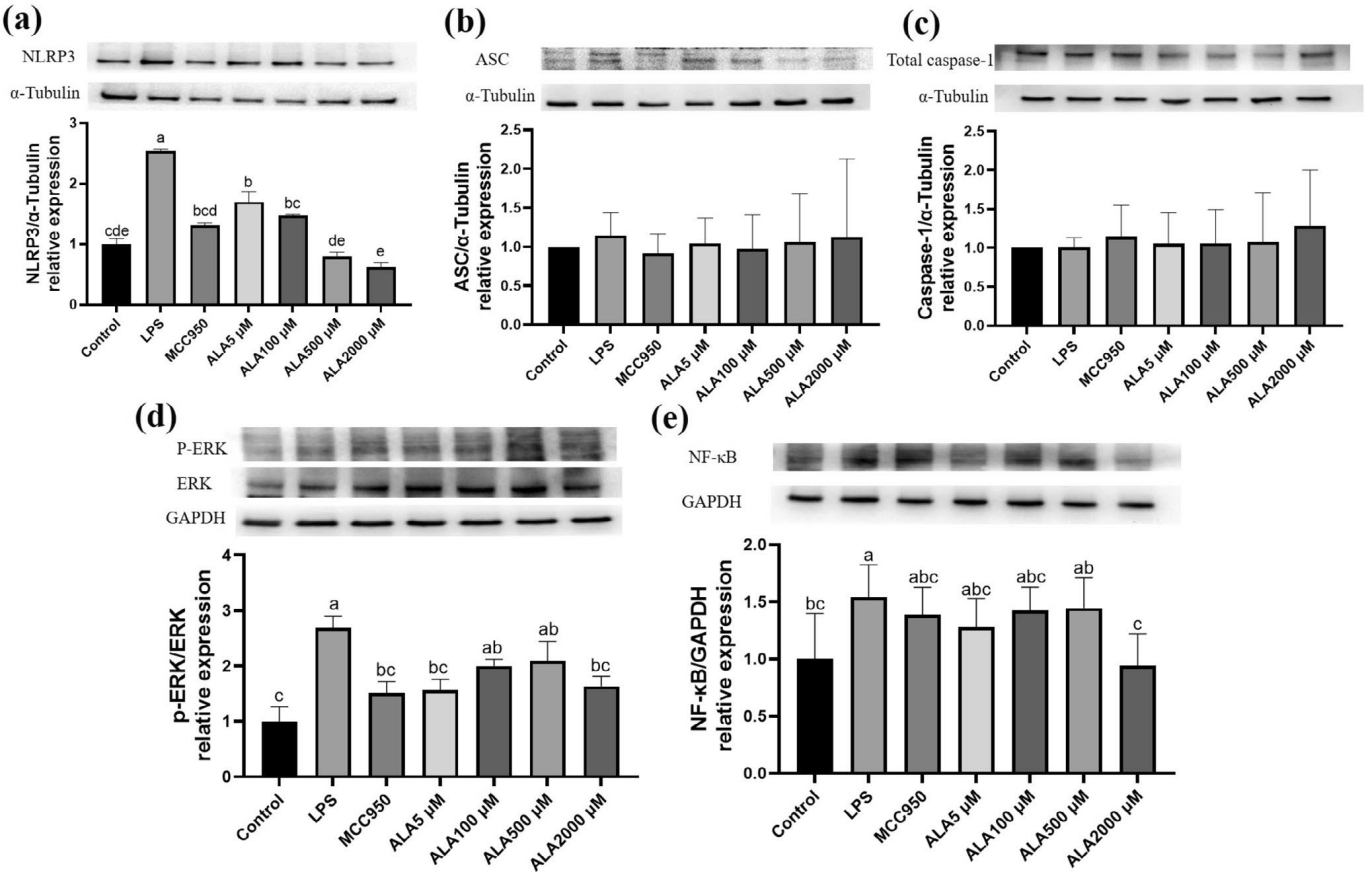
Effect of alpha‐lipoic acid (ALA) on NLRP3 inflammasome–related protein expression in Kupffer cells following LPS priming and ATP stimulation. Representative immunoblots and densitometric quantification of NLRP3 (a), ASC (b), total caspase‐1 (c), phosphorylated ERK/ERK (d), and NF‐κB (e). Data are presented as mean ± SD, *n* = 3 for each group. a‐e letter is significantly different among all samples tested (*p* < 0.05).

Kupffer cells stimulated with LPS and ATP exhibited a significant increase in NLRP3 expression, with a 254.2% elevation compared to the control group. Pretreatment with either MCC950 (20 μM) or ALA (5–2000 μM) markedly suppressed NLRP3 expression; the most pronounced reduction was observed with 2000 μM ALA, which reduced to 191.7% of the LPS group (*p* < 0.05) (Figure 4a). The expression of ASC modestly increased by 14.1% following LPS exposure and was slightly reduced by treatment with 100 μM ALA and MCC950, while other ALA concentrations did not significantly affect ASC expression (Figure 4b). Total caspase‐1 expression remained unchanged across all experimental groups (Figure 4c). Additionally, LPS stimulation elevated nuclear factor‐kappa B (NF‐κB) expression by 44.5%, which was significantly downregulated by pretreatment with 2000 μM ALA (*p* < 0.05) (Figure 4d). The ratio of phosphorylated extracellular signal‐regulated kinase (p‐ERK) to total ERK increased by 109.1% in response to LPS, but this effect was attenuated in a dose‐dependent manner by ALA (Figure 4e). These findings suggest that ALA attenuates inflammasome‐related signaling/priming markers and modulates upstream inflammatory pathways in Kupffer cells.

### 3.5 ALA Pretreatment of Kupffer Cells Enhances Glucose Uptake in Hepatocytes Cultured With Conditioned Medium From LPS‐Primed/ATP‐Stimulated Kupffer Cells

**FIGURE 5:**
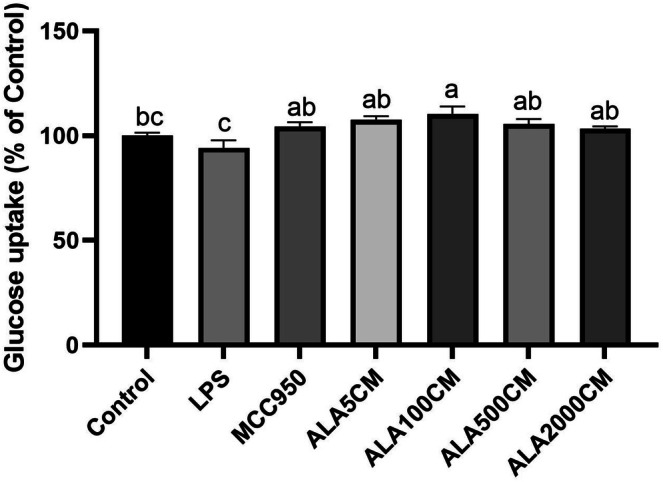
Effect of alpha‐lipoic acid pretreatment in Kupffer cells on glucose uptake in FL83B hepatocytes exposed to conditioned medium from Kupffer cells that were primed with LPS and subsequently stimulated with ATP. ALA5CM, ALA100CM, ALA500CM, and ALA2000CM: Conditioned media collected from LPS‐primed/ATP‐stimulated Kupffer cells that were pretreated with alpha‐lipoic acid (ALA) at concentrations of 5, 100, 500, or 2000 μM, respectively, for 6 h prior to LPS stimulation. Data are presented as mean ± SD, *n* = 4 for each group. a‐c letter is significantly different among all samples tested (*p* < 0.05).

To assess the impact of Kupffer cell‐derived inflammatory signals on hepatocyte insulin responsiveness, FL83B hepatocytes were incubated with conditioned medium. The glucose uptake was significantly reduced in FL83B cells cultured with conditioned medium obtained from LPS‐primed/ATP‐stimulated Kupffer cells, indicating impaired insulin sensitivity. In contrast, Kupffer cells pretreated with ALA significantly restored glucose uptake (*p* < 0.05) in FL83B hepatocytes cultured with conditioned medium from LPS‐primed/ATP‐stimulated Kupffer cells, suggesting an improvement in insulin responsiveness (Figure 5).

### 3.6 Pretreatment of Kupffer Cells With ALA Prior to LPS Stimulation Improves Insulin Signaling in Hepatocytes Cultured With Conditioned Medium From LPS‐Primed/ATP‐Stimulated Kupffer Cells

**FIGURE 6:**
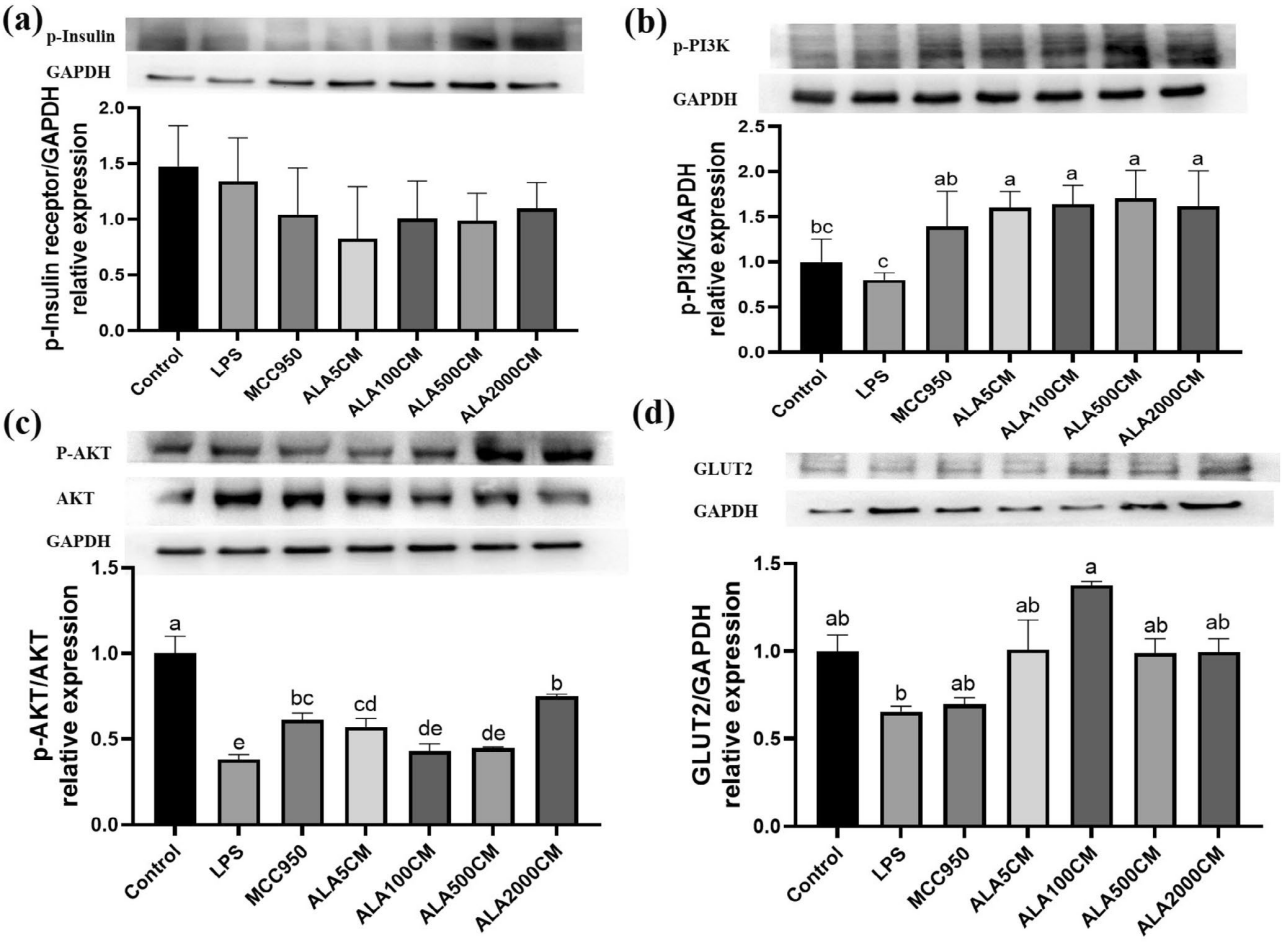
Effect of alpha‐lipoic acid pretreatment in Kupffer cells on insulin signaling in FL83B hepatocytes exposed to conditioned medium from LPS‐primed followed by ATP stimulating Kupffer cells. Representative immunoblots and densitometric quantification of phosphorylated insulin receptor (a), phosphorylated PI3K (b), phosphorylated AKT (p‐AKT)/AKT (c), and GLUT2 (d). ALA5CM, ALA100CM, ALA500CM, and ALA2000CM: Conditioned media collected from LPS‐primed/ATP‐stimulated Kupffer cells that were pretreated with alpha‐lipoic acid (ALA) at concentrations of 5, 100, 500, or 2000 μM, respectively, for 6 h prior to LPS stimulation. Data are presented as mean ± SD, *n* = 3 for each group. a‐e letter is significantly different among all samples tested (*p* < 0.05).

Western blot analysis indicated that conditioned medium from ALA‐pretreated Kupffer cells partially restored insulin signaling in FL83B hepatocytes. The p‐IR showed no statistically significant change compared with the LPS‐CM group, whereas downstream signals (p‐PI3K and p‐AKT) were increased. Importantly, GLUT2 expression was significantly upregulated in the ALA100‐CM group (*p* < 0.05) (Figure 6), that is consistent with the improved glucose uptake observed in Figure 5.

## 4 Discussion

**FIGURE 7:**
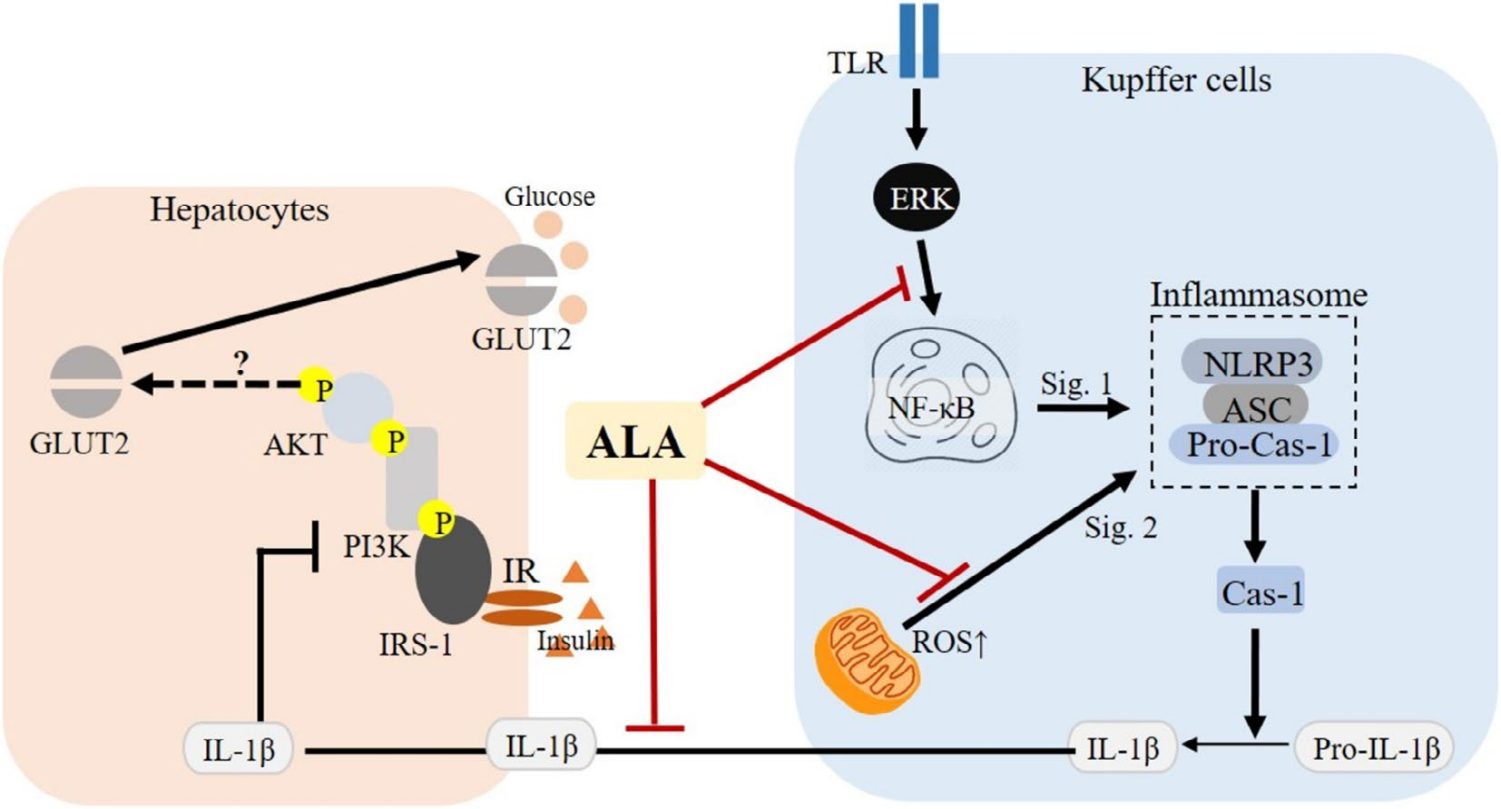
The postulated mechanism of alpha‐lipoic acid (ALA) on suppressing lipopolysaccharide (LPS)‐induced Kupffer cells inflammation and its conditioned medium‐incubated FL83B hepatocytes insulin resistance.

In this study, we demonstrated that ALA significantly attenuates inflammatory responses in LPS‐primed/ATP‐stimulated Kupffer cells and improves insulin signaling in hepatocytes exposed to their conditioned medium. The dosage of 2000 μM ALA is the most potent inhibitor of Kupffer‐cell inflammatory markers, whereas 100 μM‐derived conditioned medium confers the greatest hepatocyte benefit. Specifically, ALA pretreatment reduced IL‐1β and TNF‐α secretion, attenuated NF‐κB activation and ERK phosphorylation, and decreased NLRP3/ASC expression, while preserving MMP in Kupffer cells. As a result, FL83B hepatocytes incubated with conditioned medium from ALA‐pretreated Kupffer cells exhibited enhanced glucose uptake and upregulation of insulin signaling proteins, including p‐PI3K, p‐AKT, and GLUT2. These findings suggest that ALA might modulate the inflammatory response of hepatic immune cells, thereby restoring insulin sensitivity in hepatocytes under inflammatory conditions (Figure 7).

Kupffer cells subjected to LPS priming followed by ATP stimulation exhibited robust inflammasome‐related responses, including increased NLRP3 expression and elevated IL‐1β and TNF‐α secretion. Pretreatment with ALA significantly reduced these inflammatory markers, consistent with previous research demonstrating that ALA suppresses NLRP3 inflammasome via redox‐sensitive pathways (Rochette et al. 2015; Yang et al. 2019). Similar anti‐inflammatory effects have been observed in neuroblastoma and adipose models, where ALA inhibited IL‐1β and IL‐6 via epigenetic regulation and NF‐κB inactivation (Dinicola et al. 2017; Capece et al. 2022). The inhibition of p‐ERK and NF‐κB by ALA in our model underscores its upstream immunomodulatory capacity.

Furthermore, the preservation of MMP by ALA in Kupffer cells supports its role as a mitochondrial‐targeted antioxidant (Shay et al. 2009). Mitochondrial dysfunction and ROS overproduction are known to activate NLRP3 inflammasomes and disrupt hepatic homeostasis (Wang et al. 2019). Our findings align with those of Rochette et al. (2015), which showed ALA preserved mitochondrial health in diabetic models, likely by maintaining redox homeostasis. While we did not measure ROS levels directly, our JC‐1 assay of ALA provides indirect evidence of mitochondrial stabilization.

A key feature of this study is the use of conditioned medium from LPS‐primed/ATP‐stimulated Kupffer cells to evaluate their paracrine impact on hepatocytes. Conditioned medium from activated Kupffer cells significantly suppressed glucose uptake and reduced phosphorylation of PI3K and AKT in FL83B hepatocytes, suggesting impairment of insulin signaling. The finding echoes previous reports indicating that proinflammatory cytokines, especially IL‐1β and TNF‐α, interfere with insulin receptor pathways by inhibiting tyrosine phosphorylation and promoting serine phosphorylation (Jo et al. 2016; Meier et al. 2025).

ALA pretreatment of Kupffer cells mitigated this adverse paracrine effect, restoring insulin sensitivity in hepatocytes, as evidenced by increased expression of p‐PI3K, p‐AKT, and GLUT2. These results agree with prior work showing that ALA stimulates GLUT4 translocation and glucose uptake in muscle and adipose tissues via the PI3K‐AKT pathway (Konrad et al. 2001; Yaworsky et al. 2000). Moreover, data from diabetic rats confirm that ALA enhances insulin signaling in multiple tissues (Ko, Lo, et al. 2021; Ko et al. 2023).

Among the tested concentrations, 2000 μM ALA exerted the most potent suppression of inflammatory factors in Kupffer cells, whereas conditioned medium derived from the 100 μM ALA group conferred the greatest improvement in hepatocyte glucose uptake and GLUT2 expression, indicating a non‐linear paracrine dose–response. This pattern is consistent with previous research indicating that ALA exhibits dose‐dependent behavior: low to moderate doses act as antioxidants, while excessively high doses may paradoxically promote oxidative stress via redox cycling (Moini et al. 2002; Cakatay 2006). While 2000 μM ALA was effective in the present study, its benefits plateaued, likely due to saturation kinetics or negative feedback. This highlights the importance of dosing in therapeutic contexts and supports findings from clinical trials suggesting optimal ALA supplementation should not exceed moderate pharmacological thresholds (de Oliveira et al. 2011; Jibril et al. 2022).

Our findings extend prior in vivo studies where ALA improved hepatic steatosis, reduced inflammatory markers (Ko, Lo, et al. 2021), and enhanced glucose homeostasis in diabetic rat models (Ko et al. 2023). Notably, our work moves beyond systemic observations and dissects the intrahepatic immune‐metabolic crosstalk, offering mechanistic insights into how Kupffer cell‐derived signals modulate hepatocyte insulin sensitivity. Clinical meta‐analyses have also validated the role of ALA in improving insulin resistance and glycemic control in T2DM patients (Jibril et al. 2022), reinforcing the translational value of our findings.

This study offers several important academic contributions. First, it provides direct evidence that ALA attenuates LPS‐driven NLRP3 inflammasome–related priming/signaling markers (NLRP3/ASC expression and upstream NF‐κB/ERK signaling) and reduces IL‐1β release in Kupffer cells, which has not been fully addressed in previous hepatic models. Second, it establishes a mechanistic link between Kupffer cell‐mediated inflammation and hepatocyte insulin resistance, reinforcing the significance of immune‐metabolic crosstalk in liver physiology. Third, our dual‐cell culture system, incorporating Kupffer cells and FL83B hepatocytes, provides a relevant in vitro platform for studying hepatic paracrine interactions under inflammatory conditions. Lastly, the identification of 100 μM as the most effective ALA concentration contributes to the rational design of dosing strategies for future translational and clinical applications. Overall, these findings not only enhance our understanding of hepatic immune regulation in metabolic diseases but also support the use of ALA as a potential adjunct therapy for insulin resistance.

Despite its strengths, this study has several limitations. First, it is based entirely on an in vitro model using immortalized murine Kupffer cells and hepatocytes. While useful for mechanistic exploration, such models do not fully capture the complexity of the hepatic microenvironment in vivo, including interactions with other immune and stromal cells. Future research should validate these findings in animal models or human primary cell systems to establish physiological relevance. Second, although we demonstrated mitochondrial preservation via JC‐1 staining, we did not directly measure ROS levels, mitochondrial respiration, or redox state. Given the central role of oxidative stress in NLRP3 activation, these parameters should be included in future studies to confirm ALA's antioxidant mechanism. Third, the current work focused mainly on IL‐1β and TNF‐α. Other key mediators, such as IL‐6, monocyte chemoattractant protein‐1, or the involvement of NF‐κB and JNK pathways could also contribute to the observed effects and merit deeper investigation. Moreover, we did not assess inflammasome assembly via ASC speck formation or caspase‐1 cleavage, which would strengthen our conclusions regarding inflammasome suppression. In the present study, ALA markedly attenuated NF‐κB activation and reduced NLRP3 and ASC levels, both of which are essential for formation of the NLRP3‐ASC‐caspase‐1 complex. According to the canonical inflammasome pathway, diminished priming (pro‐IL‐1β, NLRP3) and impaired complex assembly are expected to limit autocatalytic processing of pro‐caspase‐1 into its active (cleaved) form and thereby decrease conversion of pro‐IL‐1β to mature IL‐1β, even if total caspase‐1 protein remains unchanged (Guo et al. 2015; Broz and Dixit 2016). Lastly, the therapeutic implications of our findings require confirmation in clinical settings. Although ALA is already used in diabetic neuropathy and shows promise in glycemic control, optimal formulations, delivery methods, and combination therapies remain to be defined. Future studies should evaluate long‐term safety, dosage optimization, and combinatorial approaches with existing antidiabetic agents.

## 5 Conclusions

The present study demonstrates that pretreatment of Kupffer cells with ALA reduces inflammatory responses, attenuates NLRP3 inflammasome–related priming/signaling markers, decreases IL‐1β secretion, and helps maintain mitochondrial integrity following LPS stimulation. Moreover, conditioned medium derived from these ALA‐treated Kupffer cells alleviates insulin resistance in FL83B hepatocytes, as indicated by increased glucose uptake and enhanced activation of the PI3K and AKT signaling cascade. These findings offer mechanistic insight into how hepatic immune responses influence insulin sensitivity and suggest that ALA might serve as a promising therapeutic compound for managing metabolic disorders associated with chronic inflammation and impaired insulin signaling. Nevertheless, further validation through in vivo experimentation and clinical studies is essential to confirm the physiological significance, safety profile, and translational potential of ALA in the treatment of metabolic diseases.

## Author Contributions

**Chung‐Hsin Wu:** investigation, writing – review and editing. **Chih‐Yuan Ko:** writing – original draft, writing – review and editing, software, validation, conceptualization, investigation, visualization, funding acquisition. **Szu‐Chuan Shen:** conceptualization, investigation, writing – original draft, funding acquisition, writing – review and editing, project administration, supervision, resources. **Yangming Martin Lo:** conceptualization, investigation, writing – original draft, writing – review and editing. **Thi Kim Ngan Nguyen:** writing – original draft, writing – review and editing. **Shao‐Ting Kao:** investigation, methodology, visualization, writing – original draft. **Wen‐Chung Huang:** writing – original draft, writing – review and editing.

## Funding

This work was supported by the Ministry of Science and Technology of the Republic of China (ROC) under Grant Number MOST 107‐2320‐B‐003‐004‐MY3, the National Natural Science Foundation of China (no. 82541160283), the Natural Science Foundation of Fujian Province (no. 2025J01835), and Joint Funds for the innovation of science and Technology, Fujian Province (no. 2025Y9423).

## Conflicts of Interest

The authors declare no conflicts of interest.

## Data Availability

The data that support the findings of this study are available from the corresponding author upon reasonable request.
